# Stable synthesis of few-layered boron nitride nanotubes by anodic arc discharge

**DOI:** 10.1038/s41598-017-03438-w

**Published:** 2017-06-08

**Authors:** Yao-Wen Yeh, Yevgeny Raitses, Bruce E. Koel, Nan Yao

**Affiliations:** 1 Princeton Plasma Physics Laboratory, Princeton, New Jersey 08543 USA; 2 Princeton Institute for Science and Technology of Materials (PRISM), Princeton University, Princeton, New Jersey 08544 USA; 3 Department of Chemical and Biological Engineering, Princeton University, Princeton, New Jersey 08544 USA

## Abstract

Boron nitride nanotubes (BNNTs) were successfully synthesized by a dc arc discharge using a boron-rich anode as synthesis feedstock in a nitrogen gas environment at near atmospheric pressure. The synthesis was achieved independent of the cathode material suggesting that under such conditions the arc operates in so-called anodic mode with the anode material being consumed by evaporation due to the arc heating. To sustain the arc current by thermionic electron emission, the cathode has to be at sufficiently high temperature, which for a typical arc current density of ~100 A/cm^2^, is above the boron melting point (2350 K). With both electrodes made from the same boron-rich alloy, we found that the arc operation unstable due to frequent sticking between two molten electrodes and formation of molten droplets. Stable and reliable arc operation and arc synthesis were achieved with the boron-rich anode and the cathode made from a refractory metal which has a melting temperature above the melting point of boron. *Ex-situ* characterization of synthesized BNNTs with electron microscopy and Raman spectroscopy revealed that independent of the cathode material, the tubes are primarily single and double walled. The results also show evidence of root-growth of BNNTs produced in the arc discharge.

## Introduction

Recently, much research has been devoted to boron nitride nanotube (BNNT) synthesis as they possess attractive properties such as high Young’s modulus^1, 2^, resistance to oxidation^3^, and high absorption energy for hydrogen molecules^4, 5^. These properties permit BNNTs to be used in not only structurally reinforced composites but also hydrogen storage applications^6^. Similar to carbon nanotubes (CNTs), BNNTs can be synthesized by plasma-based approaches^1, 7, 8^, laser ablation^9, 10^, chemical vapor deposition (CVD)^11^, and ball milling^12^. Among possible synthesis routes reported to date, plasma-based approaches and laser ablation remain attractive synthesis techniques as they are able to produce high quality BNNTs almost instantaneously. Among the plasma-based approaches, near-atmospheric pressure dc arc discharges^1, 13^, atmospheric pressure and high pressure RF plasma torches^7, 8^ were the only successful methods that synthesized BNNT in volume. From a practical standpoint, the main advantage of arc discharge synthesis is that it requires a very simple and inexpensive setup compared to plasma torch reactors^7, 8^.

Since the discovery of fullerene^14^, many technologically important nanomaterials such as carbon nanotubes (CNTs)^15–17^, boron nitride nanotubes (BNNTs)^1, 18–21^, boron carbon nitride^22^, zinc oxide^23^, and gallium oxide nanowires^24^ have been synthesized by arc plasmas. However, material growth mechanisms in arc plasmas remain elusive due to the extreme condition associated with arc plasmas even for the widely studied carbon arc. In our recent studies^16, 17^, we demonstrated a crucial role of the carbon cathode deposit in the operation of a typical atmospheric pressure carbon arc for synthesis of multi-walled and single-walled CNTs. Essentially, this arc cannot operate without the carbon deposit produced from the ablated products of the graphite anode. The ablation of the graphite anode by the arc discharge serves also as the synthesis feedstock of carbon atoms and molecules. For arc synthesis of BNNTs, the formation of the cathode deposit from dielectric boron nitride materials would imply the interruption of the dc arc current. This poses a key challenge for the use of dc arc discharges for the synthesis of BNNTs and other non-conductive materials.

Previous studies of the arc discharge for synthesis of BNNTs did not address stability of the arc operation and the synthesis processes. Early studies of arc synthesis of BNNTs have been reported with various boron containing electrodes such as HfB_2_^18^ and ZrB_2_^19^ in nitrogen gas environments and boron nitride encapsulated composite electrode^21^ in helium gas environments. Yet in these studies BNNTs were only found in the cathode deposits. Until now, there have been only two reports of successful arc synthesis of BNNTs in volume^1, 13^. The arc was operated with anode and cathode electrodes made from the same boron-rich alloy in a nitrogen gas filled chamber at 380 torr. After operation of this arc, a grey web-like material consisting of BNNTs was found in the reactor chamber suggesting a volumetric synthesis of BNNTs. However, among the materials synthesis arcs reported to date, the arcs are mostly anodic and the synthesis feedstock comes from the anodes except the arc used to produce BNNTs. Presumably, the purpose of using the same boron-containing materials for both cathode and anode is to increase the likelihood of BNNT production since both electrodes receive a significant amount of heat from the arcs. Nevertheless, considering the cost of these specialized electrodes, it is worth exploring the necessity of using the boron-containing cathode.

In this work, we report the successful volumetric synthesis of BNNTs by the arc discharge and the evidence of root-growth mechanism of BNNTs. To the best of our knowledge, we report herein the first image as evidence for root-growth of BNNTs from arc synthesis. This evidence is similar to the one reported in the laser ablation based BNNT synthesis^25^, which indicates that the root-growth mechanism may be one universal growth mechanism in high temperature BNNT syntheses. We also demonstrate that the evaporation of the anode material provides a synthesis feedstock of boron, while a relatively high voltage of the arc discharge in the nitrogen arc should be sufficient to sustain a synthesis feedstock of nitrogen atoms produced by dissociation of nitrogen molecules. Finally, it is also shown that the use of refractory metals as the cathode material as opposed to the boron-rich alloy allows to achieve more stable and continuous operation of the arc and synthesis of BNNTs.

## Results

### Anodic discharge

BNNTs were synthesized by the arc discharge operated with a discharge current of 40 A and discharge voltage of 35 to 40 V at 400 torr of N_2_. A more detailed description of arc operation is described in Methods. One of the crucial requirements to successful arc synthesis is to have conductive electrodes that are able to sustain the dc current in the electric circuit of the arc. Boron is a semiconducting material that does not have enough conductivity to initiate the arc at room temperature^1, 26^. In order to overcome this issue, trace amounts of nickel and cobalt were incorporated in the boron electrodes such that the arc could be ignited without an additional heating mechanism. It was found that the boron anode was consumed and participated in boron nitride synthesis reaction with background nitrogen gas, while the cathode materials could be either tungsten or boron without affecting the synthesis results. After the discharge, grey fibrous products were found on the electrodes, a material collector sheet placed under the electrodes, and chamber wall without a preferred deposit location. Due to the spatially random deposition of the fibrous products, it is difficult to collect all the products and accurately characterize the synthesis yield. This result is similar to the case of metal-catalyst-assisted arc production of single walled CNTs, in which a black web-material containing the nanotubes can also be found on the chamber walls and electrodes. It is commonly accepted that this web material is a manifestation of the volume growth nanotubes. In addition to the carbon web, a carbon deposit with multiwalled nanotubes grows on the cathode. During the carbon arc operation, this deposit acts as an effective thermionic emitter, which is heated by the heat flux from the plasma due to thermal conduction, ion bombardment, and the latent heat brought to the cathode surface with ablated products from the graphite anode^16, 17, 27^. However, unlike the case of the carbon arc, there is nearly no deposit on the plasma-facing tip of the tungsten cathode. As will be discussed later, during the arc operation, the tungsten cathode tip is heated to temperatures of above 3000 K.

**Figure 1:**
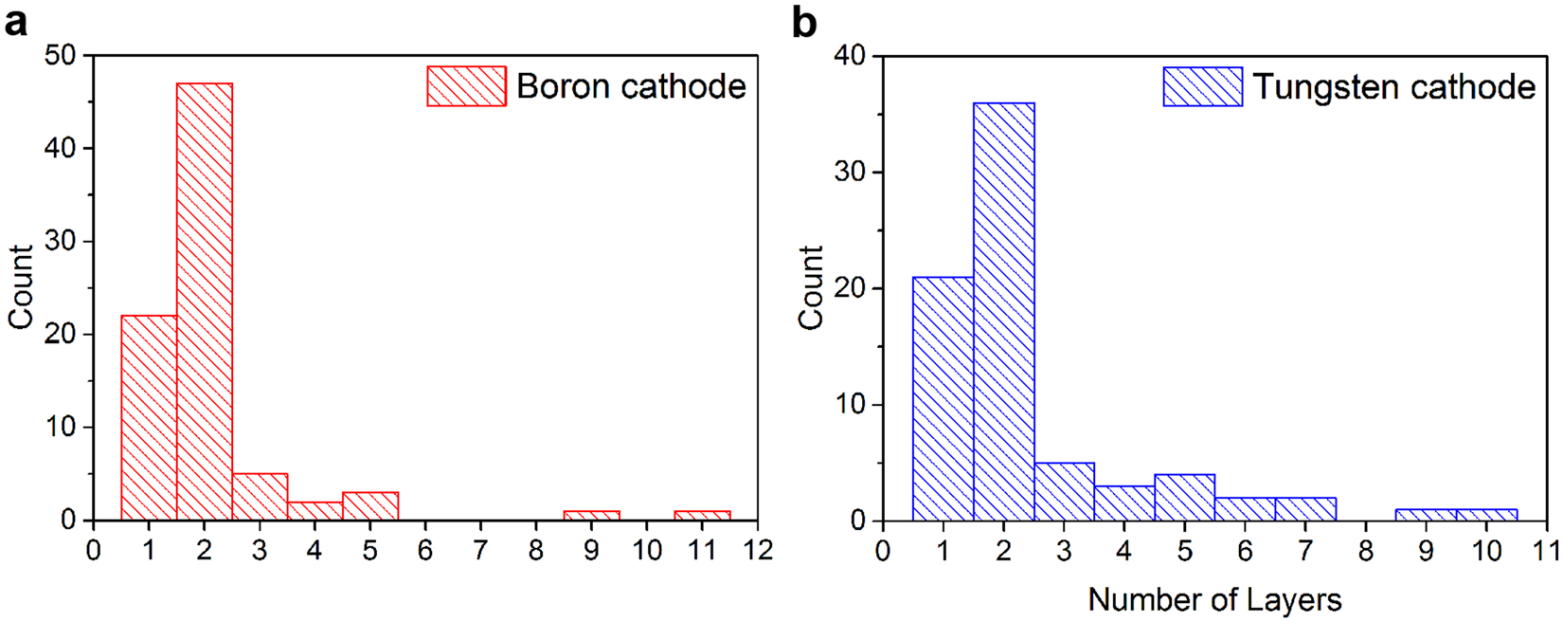
BNNTs synthesized with (**a**) boron and (**b**) tungsten cathodes show similar results. The tubes were primarily single and double walled.

**Figure 2:**
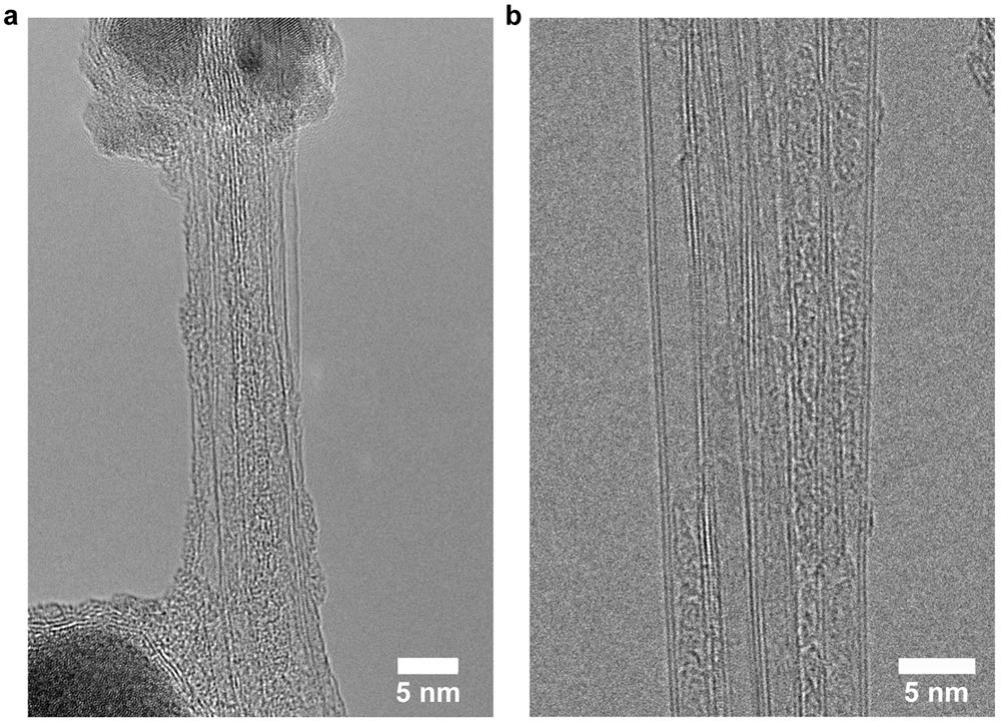
(**a**) Single- and (**b**) double-walled BNNT bundles.

The fibrous products synthesized with tungsten and boron cathodes were surveyed by TEM. The typical appearance of the fibrous products is shown in Fig. S1. In both cases, the results were nearly identical, and the products contained BNNTs along with unreacted anode material wrapped by layers of boron nitride. As shown in Fig. 1, the BNNTs synthesized by the arc are primarily single and double walled, with no affect by the cathode materials. Both the single- and double-walled nanotubes are typically bundled as shown in Fig. 2, and their lengths are a few micrometers long, which is similar to the lengths reported by Cumings and Zettl^1^. This observation indicates that the tungsten cathode did not affect the BNNT synthesis and that the discharge is anodic, which is similar to the discharge for CNT synthesis by arc.

In addition, the discharge was stable and could be run continuously for minutes without interruption when a tungsten cathode was used. A stable arc operation would lead to better synthesis throughput. Together with the TEM survey, this observation indicates that the discharge is of an anodic nature where the cathode’s primary role is to provide electron emission that supports the arc current.

**Figure 3:**
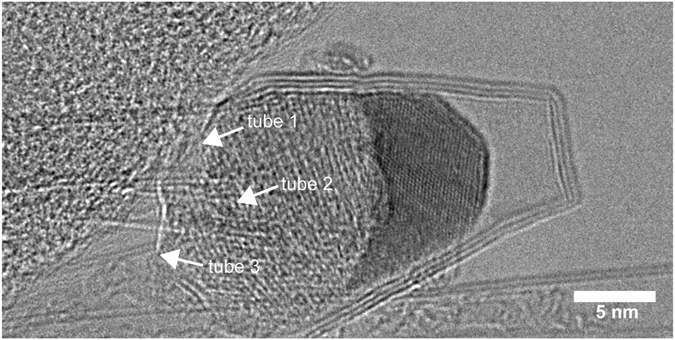
Three BNNTs grown out of an unreacted anode nanoparticle. The particle is also wrapped by boron nitride layers.

Occasionally, tube ends were located during the TEM survey. It was observed that nanotubes could grow from boron anode material that did not react fully. Figure 3 shows there are three nanotubes grown out of a boron nanoparticle, which the observation suggests a root-growth mechanism of the nanotubes. The particle shows two regions of different contrasts indicating that the particle is composed of dissimilar elements, which will be discussed in material characterization section.

### Cathode temperature

**Figure 4:**
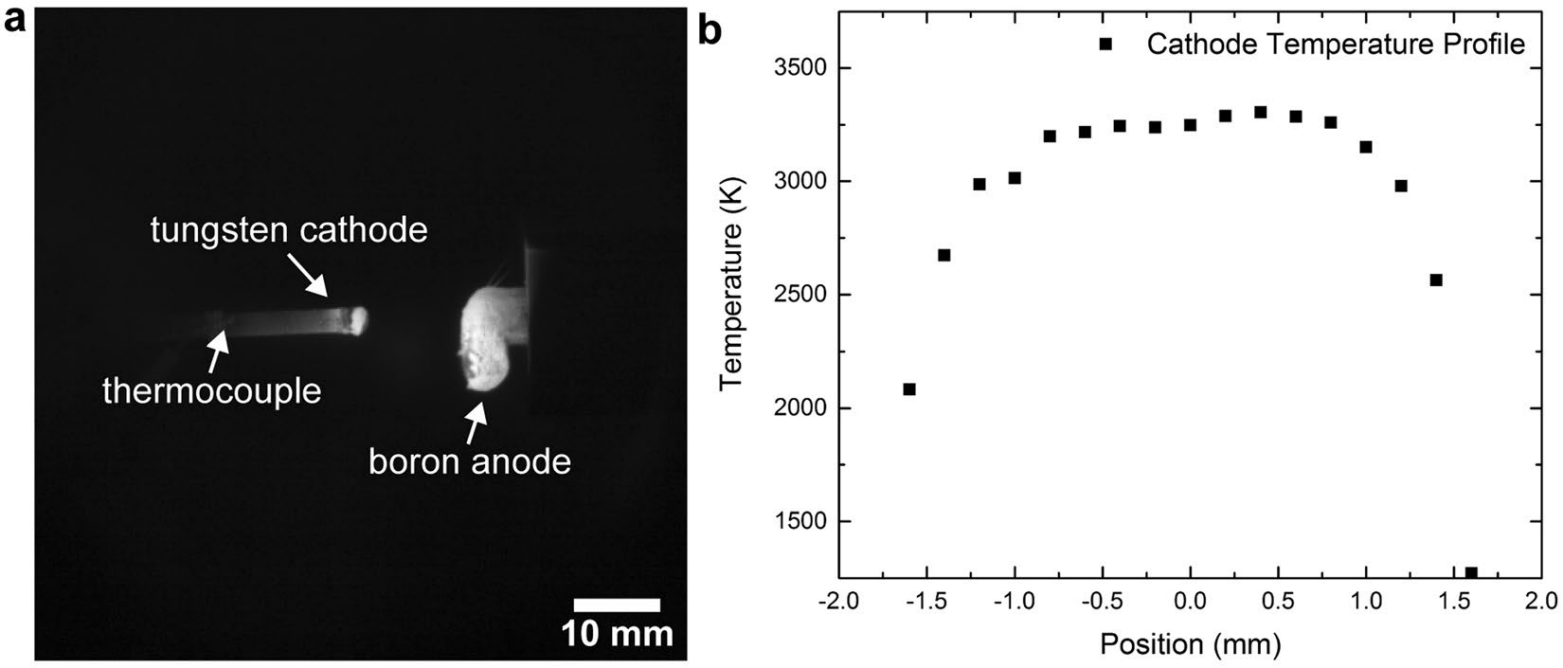
(**a**) IR image of the electrodes during the arc discharge. (**b**) Temperature profile of the plasma facing tungsten cathode tip. A position of zero corresponds to the center along the width of the electrode (3.2 mm dia.), with other relative positions toward the electrode edge indicated.

During the discharge, the temperature of the tungsten cathode was monitored by an IR camera as described in Methods. The camera is calibrated by a C-type thermocouple attached to the tungsten cathode as shown in Fig. 4a. We observed that the boron anode melted and deformed during the discharge, while the tungsten cathode retained its shape. Taking the temperature data derived from the IR images, the surface temperature profile across the plasma facing tungsten tip is plotted as shown in Fig. 4b. The temperature of the cathode tip could be as high as 3200 K during the discharge. The corresponding thermionic electron emission from the cathode can be estimated by:

$$J={A}_{0}{T}^{2}{e}^{\frac{-W}{kT}}$$

where J is the electron current density, A_0_ is Richardson constant (120 A∙mm^−2^∙K^−2^), T is the temperature (K), W is the work function of tungsten (4.55 eV), and k is the Boltzmann constant (8.62 × 10^−5^ eV∙K^−1^). We found that thermionic emission of electrons from the hot 10 mm^2^ area of the cathode tip could support the arc current (40 A).

It should be noted that while boron has a work function of 4.45 eV^28^, similar to that of tungsten, boron melts at 2350 K, which is below the temperature required to support the arc current thermionically. While the exact contributions of electrons and ions to the total current at its attachment to the cathode are unknown, it is reasonable to assume that for the same arc current and nitrogen pressure, they are the same for both boron-rich cathode and tungsten cathodes. This implies that the temperature of the cathodes should also be the same in order to sustain the same thermionic electron emission current. However, unlike tungsten, the boron-rich cathode has to be in a molten state at 3200 K. In the arc operation with two molten boron-rich electrodes, we observed their frequent sticking due to the formation of molten droplets. Consequently, this electrode sticking leads to interruption of the arc discharge and decreases the synthesis throughput.

### Material characterization

**Figure 5:**
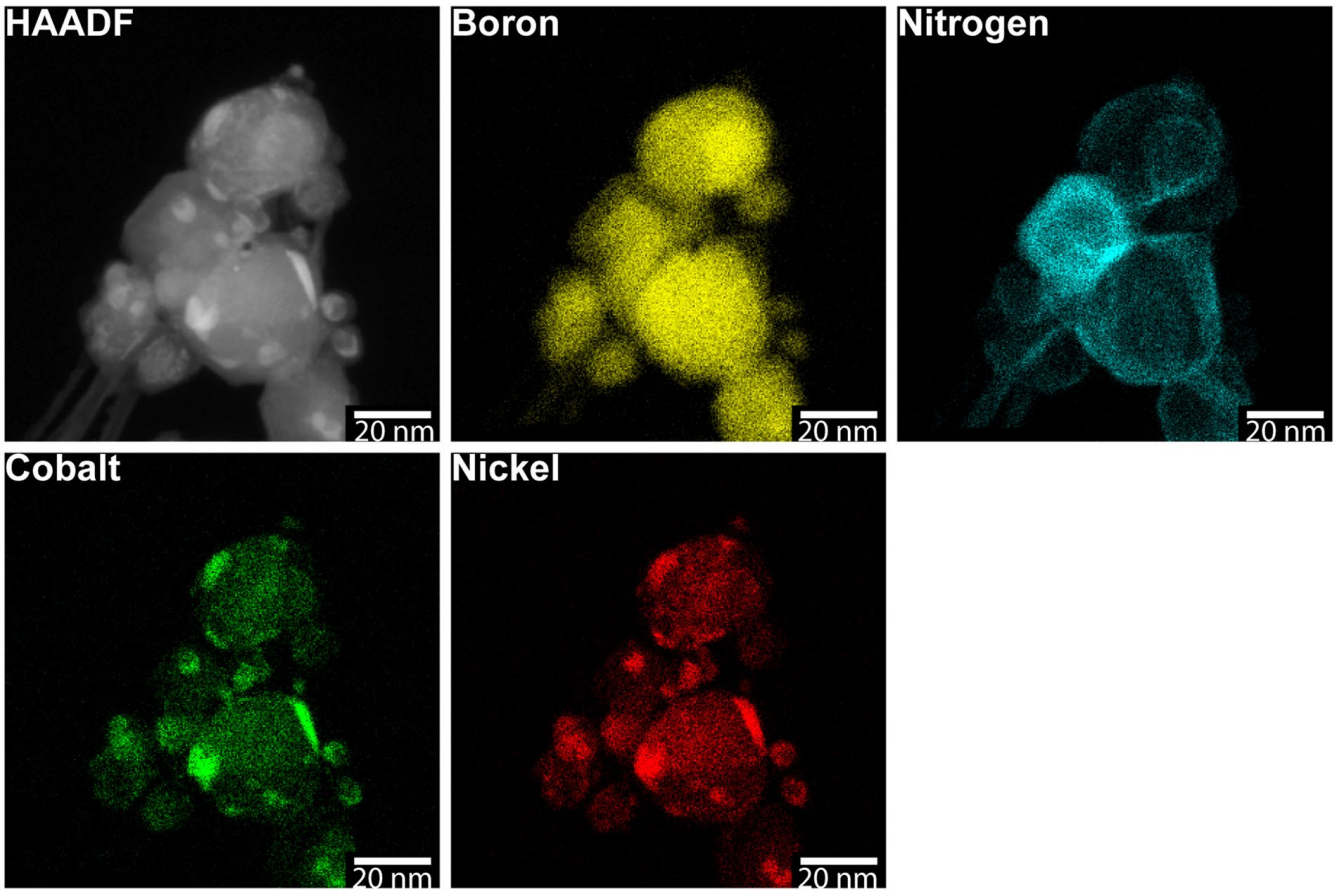
EDS elemental mapping results. The particles contain boron, cobalt, and nickel, characteristic of the starting anode material used in the arc synthesis. Boron and nitrogen mapping results show that the tube bundles on the lower left are boron nitride and the nanoparticles are wrapped by boron nitride.

To validate the nanotubes synthesized by the arc discharge are boron nitride, the synthesized materials were characterized by energy dispersive X-ray spectroscopy (EDS) and Raman spectroscopy. EDS is useful for identifying the elements contained in the nanoparticles and confirming that the layered structures are made of boron and nitrogen. We used a scanning transmission electron microscope (STEM) fitted with EDS for these measurements. One can determine the presence of boron in the nanostructures via EDS, but it is difficult to quantify the amount of boron due to the low X-ray energy. Along with the nanotubes, there are particles produced ranging in size from 5 to 50 nm. As shown in Fig. 5, these nanoparticles are unreacted anode material that contains boron, cobalt, and nickel. The synthesized nanotubes contained only boron and nitrogen, without any cobalt or nickel trapped in nanotubes; both cobalt and nickel were only found in the unreacted anode particles. In addition, these particles were wrapped by layers of boron nitride similar to the case shown in Fig. 3. The observation of nickel and cobalt raises a question: is a metal catalyst(s) needed for boron nitride nanotube growth? For the RF plasma torch based BNNT syntheses reported by Fathalizadeh *et al*.^7^ and Kim *et al*.^8^, no metal catalyst was required to produce BNNTs. On the other hand, in the arc discharge BNNT synthesis reported by Cumings and Zettl^1^, they could not be certain about the role of cobalt and nickel in the tube growth. In order to investigate this question in the case of our present arc discharge approach, we carried out separate arc discharge experiments that used pure boron as the starting material, and we still produced boron nitride nanotubes. The results indicate that a metal catalyst(s) is not required for the tube growth. The root-growth evidence similar to Fig. 3 was also observed, but the particle contains only boron, which this on-going work will be the subject of another paper to be reported separately.

**Figure 6:**
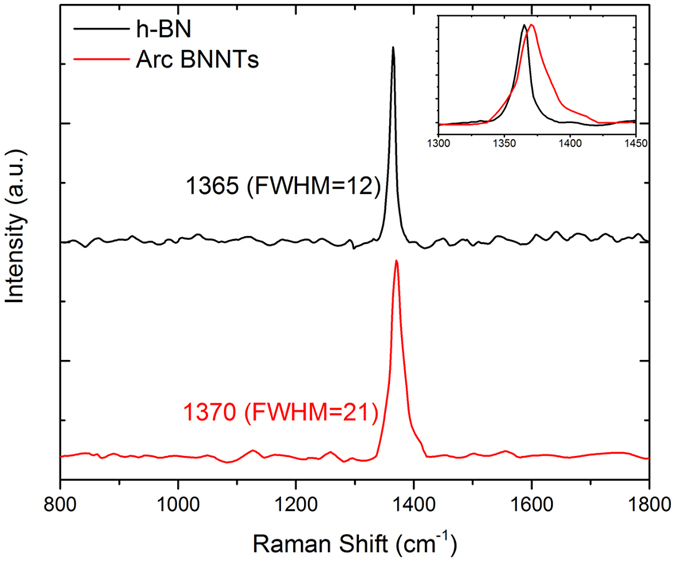
Rama spectra of bulk hexagonal boron nitride powder (black) and the arc synthesized BNNTs (red). Only the E_2g_ mode of hexagonal boron nitride was observed, and the blue shift observed in the synthesized BNNTs is characteristics of few-layer boron nitride.

Raman spectroscopy was employed to confirm that the boron and nitrogen found in the product corresponds to hexagonal boron nitride (h-BN). As shown in Fig. 6, the E_2g_ mode (~1365 cm^−1^) associated with h-BN^29^ was observed from the synthesized BNNTs, with a blue-shift of 5 cm^−1^. This blue-shift can only be observed in few-layered h-BN, and this arises from a reduced interaction of neighboring sheets^30, 31^. The Raman results confirm that the synthesized products are not only h-BN, but also of few layers, which agrees with the TEM observation that the tubes are of mostly single and double walled.

## Conclusions

Arc synthesis of boron nitride nanotubes involves the ablation of the boron anode where the evaporated boron then reacts with the background nitrogen. The grey fibrous products contained an abundant amount of BNNTs along with unreacted anode materials wrapped by layers of boron nitride. These BNNT-containing grey fibrous products were found everywhere in the reactor, which is similar to the case of arc-synthesized single walled carbon nanotubes and indicates that BNNTs were synthesized in the volume. Characterization of these products by TEM, EDS, and Raman spectroscopy determined that the synthesized nanotubes were primarily single and double walled. In addition, root-growth evidence of BNNTs was observed by high resolution TEM.

We have also shown that either boron or tungsten cathodes can be used for the discharge without affecting the synthesis results. This establishes that BNNTs can be synthesized without using the same materials for both cathode and anode, which indicates that the arc synthesis of BNNTs can be by anodic discharge similar to the case of CNTs. Furthermore, measurements of the tungsten cathode temperature showed that thermionic electron emission could potentially support all the arc current. Unlike the boron-rich alloy, the use of a tungsten cathode permits sufficient electron emission without melting. We explained that the observed stable arc operation using a tungsten cathode is due to the suppression of electrode sticking.

## Methods

### Arc plasma synthesis

BNNTs were synthesized by dc arc discharge. Details of the experimental setup can be found elsewhere^16, 27^. The boron electrodes used throughout the synthesis experiments were prepared by mixing 96% B, 2% Co and 2% Ni (at.%) in an arc melting furnace at Ames Materials Preparation Center. These boron electrodes lack a well-defined shape and are about 10-mm wide, 5-mm deep and 100-mm long. For BNNT synthesis, the arc plasma was generated in a pure nitrogen environment at 400 Torr of N_2_ by briefly bringing the boron or tungsten cathode and the boron anode in contact, after which the current was maintained at 40 A. An external control system increased the electrode gap until the specified discharge voltage was reached. The gap between the electrodes did not exceed 1 cm wide. Throughout all experiments described in this paper, the voltage measured across the two electrodes was maintained at 35 to 40 V. This voltage includes the voltage drop across the arc and along the electrodes. It is typical for short gas discharges, including high current atmospheric pressure arcs, that the largest fraction of the applied voltage drops in the near cathode region^32^. Since the voltage drop across the hot electrodes does not exceed 5 V, the arc voltage of 30 V should be enough to sustain dissociation of nitrogen molecules and ionization of resulting nitrogen atoms in the near cathode region^33^. Apparently, this process is sufficient to provide a feedstock of nitrogen atoms for the synthesis of BNNTs.

### IR camera calibration

The infrared images of the electrodes during the arc experiments were acquired by a FLIR Tau 2 camera. Flux-to-temperature calibration of the camera was achieved by attaching a C-type thermocouple to the tungsten cathode within the camera’s field of view. The pixel intensities corresponding to the thermocouple were correlated to the temperatures measured via Plank’s law.

### Analytical electron microscopy characterization

The morphology of the synthesized products was first surveyed by using an FEI Talos Transmission Electron Microscope (TEM) operated at 200 kV. The chemical analysis of the products was carried out using a Bruker Energy-dispersive X-Ray Spectroscopy (EDS) system attached in the same microscope under scanning TEM mode.

### Raman spectroscopy characterization

Raman spectra of the synthesized products and boron nitride powder as a reference were acquired by a Horiba LabRam micro-Raman system. The samples were excited by a UV laser at 325 nm through a 40x objective lens. Using a 2400 l/mm grating, the spectral resolution was 6 cm^−1^. The laser power was selected such that no heating related red-shift was observed in the spectra.

## Electronic supplementary material

Supplementary Information
