# Supplementary material for: Stable synthesis of few-layered boron nitride nanotubes by anodic arc discharge
Source: Sci Rep. 2017 Jun 8;7:3075. doi: 10.1038/s41598-017-03438-w (PMC5465186; doi:10.1038/s41598-017-03438-w)

Supplementary Information

Stable synthesis of few-layered boron nitride nanotubes by anodic arc discharge

Yao-Wen Yeh, Yevgeny Raitses, Bruce E. Koel, and Nan Yao

Supplementary Fig. S1: BNNT containing fibrous products deposited on the material collector sheet placed under the electrodes during synthesis experiments.

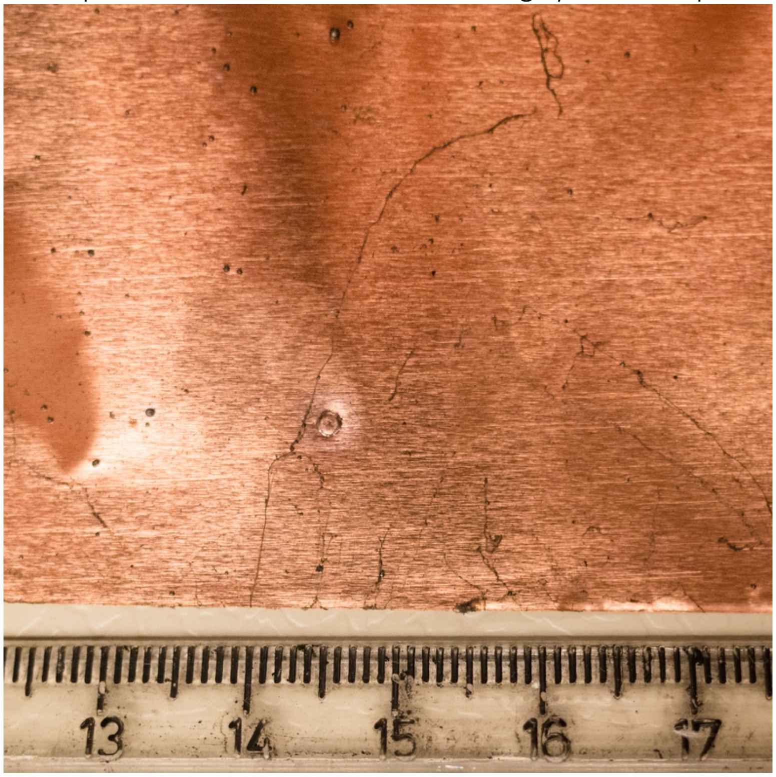

Supplement: Supplementary file 1 — Supplementary Information [file 41598_2017_3438_MOESM1_ESM.pdf]
